# Secondary alveolar cleft grafting using autogenous mineralized plasmatic matrix (MPM) versus cancellous bone particles derived from anterior iliac crest

**DOI:** 10.1007/s00784-023-05042-x

**Published:** 2023-05-05

**Authors:** Mohammed Omara, Louai Raafat, Tarek Elfaramawi

**Affiliations:** 1 Oral and Maxillofacial Surgery Department, Faculty of Dentistry, Cairo University, 11 Saraya Street, Manial, Cairo, Egypt; 2 School of Dentistry, Newgiza University, Giza, Egypt

**Keywords:** Alveolar cleft grafting, Autogenous mineralized plasmatic matrix, Sticky bone, Mixed dentition phase

## Abstract

**Objective:**

The essential concern of alveolar cleft grafting in patients of cleft lip and palate at the mixed dentition phase is to gain bone within the cleft area that provides closure of the oronasal communication with continuous and stable maxilla for future cleft teeth eruption or implantation. This study aimed to compare the effectiveness of mineralized plasmatic matrix (MPM) versus cancellous bone particles harvested from anterior iliac crest in secondary alveolar cleft grafting.

**Patients and methods:**

This prospective randomized controlled trial was conducted on ten patients with unilateral complete alveolar cleft requiring cleft reconstruction. Patients were randomly divided into two equal groups; group (1) included 5 patients who received particulate cancellous bone derived from anterior iliac crest (control group) and group (2) included 5 patients who received MPM graft prepared from cancellous bone derived from anterior iliac crest (study group). All patients received CBCT preoperatively, immediately postoperatively and after 6 months. On the CBCT, graft’s volume, labio-palatal width, and height were measured and compared.

**Results:**

The outcome of the studied patients 6 months postoperatively showed that the control group had significant decrease in the graft volume, labio-palatal width, and height compared to the study group.

**Conclusion:**

MPM allowed for the integration of bone graft particles inside a fibrin network, which offers positional stability of the bone particles, thus preserving their shape with subsequent “in situ” immobilization of the graft components. This conclusion was reflected positively in terms of maintained graft volume, width, and height compared to that of the control group.

**Clinical relevance:**

MPM allowed for maintenance of grafted ridge volume, width, and height.

## Introduction

Alveolar cleft grafting is one of the critical steps in the management of patients with cleft lip and palate. Since the twentieth century Boyne and Sands described the most common and currently utilized technique for alveolar cleft grafting. Boyne (1970) presented the concept of secondary alveolar bone grafting (SABG) which recommend the mixed dentition phase to be the preferred period of alveolar cleft grafting. Since then, several modifications for alveolar cleft grafting techniques were introduced in the literature. The main objectives of the introduced grafting techniques are gaining stability and continuity of the maxillary arch, closing of the oronasal fistula/e, preserving healthy periodontal support for the teeth adjacent to the cleft, labial and alar base support, and building the bony foundation for future implantation or eruption of the cleft teeth [1].

Bone grafting materials for alveolar cleft repair remain controversial. Different bone grafting materials were utilized including autogenic, allogenic, xenogenic, and alloplastic bone. Nevertheless, the autogenic bone remains the standard grafting material. Several donor sites for autogenic bone harvesting in alveolar cleft repair have been reported, e.g., mandibular symphysis, rib, cortical plate of cranium, tibia, and anterior and posterior iliac crest [2–4]. However, autogenous bone graft from the anterior iliac crest is considered the gold standard [5]. As it is easy to access, offers large quantities of autogenous bone which contains large amounts of osteogenic cells that promote osteogenesis and it has osteoinductive capability due to the presence of viable cells and growth factors that ensure total consolidation and incorporation with the surrounding maxillary bone [6, 7].

Many studies were published confirming the success of the gold standard technique*.* However, those studies made their evaluation based on a radiographic evaluation utilizing conventional 2D radiographs like occlusal, panoramic, or even periapical radiographs. These radiographic evaluations have their limitations and drawbacks as the distortion of 2D images, lack of reliable landmarks for measurements, superimposition from surrounding structures, and they are thought to overestimate the success of the graft as they measure only the graft height and underestimate the importance of the third dimension of the graft width labio-palataly which reflects on the total bone bridge volume. Graft width is important to determine whether the grafted bony bridge is enough to support the periodontal health of the teeth adjacent to the cleft, to support the erupting cleft canine, to support orthodontic management of the cleft teeth and for future implant placement if needed. Therefore, other imaging methods for graft assessment, such as CT and CBCT, would be superior and far more accurate for evaluating graft success from all aspects: height, width, and volume [8–17]

Many studies that evaluated the changes in the alveolar cleft bone volume and/or labio-palatal bone width after secondary alveolar cleft grafting using cancellous bone from the anterior iliac crest found that the graft volume and/or labio-palatal width overtime was not stable and was subjected to severe reduction in the total bone volume, height, and labio-palatal width [8, 9, 11, 14, 18, 19].

Different levels of the clefted maxillary segments create differences in the cleft gap width and height that hinder the proper reconstruction of the bony defect. Achievement of bone grafts of proper form and adequate stability over time remains one of the main problems. Many efforts to optimize the conformation and post-grafting results of bone derived from the anterior iliac crest have been utilized, e.g., adding barrier membranes and meshes, using a cortical piece/s of bone from the ilium either by using it as a ceiling or labio-palatal walls for the cleft gap to support the cancellous graft in between or the use of the moldable mineralized plasmatic matrix [20–28].

In addition to the moldability of the blood derived bone promoting products, for example, PRP (platelet-rich plasma), PRF (platelet-rich fibrin), and bone morphogenic protein (BMP), they are able to optimize the processes of osteogenesis, osteoinduction, and osteoconduction which are crucial for successful outcomes. Based on our experience with PRP and PRF, Mazzoni and Périssé developed MPM. MPM, containing liquid autologous platelets and fibrin concentrate, can bind to bone particles [29]. The concentrate, termed mineralized plasmatic matrix (MPM), provides better quality of the bone/fibrin mixture, resulting in a stable homogeneous material which is easy to handle. Moreover, the material expresses biologically active compounds which enhance the tissue repair mechanisms of chemotaxis, cell proliferation, angiogenesis, osteogenesis, and remodeling [30, 31]. Despite the wide use of these grafting materials, limited comparative studies had been conducted to assess its effectiveness in alveolar cleft repair compared to the conventional cancellous bone particles harvested from the anterior iliac crest. This study aimed to compare the effectiveness of mineralized plasmatic matrix (MPM) versus cancellous bone particles harvested from anterior iliac crest in secondary alveolar cleft grafting.

## Patients and methods

This was a randomized controlled clinical trial conducted on ten patients. The patients were recruited consecutively at the Department of Oral and Maxillofacial Surgery, Faculty of Dentistry, Cairo University. This study is performed in line with the principles of the Declaration of Helsinki on medical research and take a post-conduction clarification letter from the research ethics committee of the faculty of Dentistry, Cairo University. Selection of the patients was done based on the following clinical criteria: All patients were at mixed dentition phase complaining from unilateral alveolar cleft. Both sexes were included in the study. Patients must be medically and physically fit to undergo surgery under general anesthesia. Patients had no lesions at the donor and recipient sites. Whereas patients with previous attempts of alveolar cleft grafting surgery, patients with infectious, autoimmune, and/or any systemic diseases or patients with any lesions at the donor and or recipient sites were excluded from the study. Eligible patients were allocated randomly into two equal groups using a simple random sequence with an allocation ratio of 1:1 generated by a web site (www.random.org). Group (A): 5 patients with alveolar cleft in whom grafting with conventional bone particles derived from the anterior iliac crest was utilized (control group). Group (B): 5 patients with alveolar cleft in whom grafting with mineralized plasmatic matrix prepared from bone particles derived from anterior iliac crest was utilized (study group).

### Preoperative measures

Thorough medical and dental history was taken for all patients to determine the correspondence of the patients to the eligibility criteria. Written consent forms were signed by patients’ guardians/parents after clarifying the treatment plan and possible complications. A maxillary and mid-face CBCT image was captured as a baseline record using CBCT machine (Scanora® 3D with AutoSwitchTM, Soredex, Helsinki, Finland). The exposure parameters were 85 kVp, 15 Ma, and 6 cm FOV.

### Surgical procedure

All surgical procedures were performed under general anesthesia with nasotracheal intubation. Articaine (ARTINIBSA 4% 1:100000®) was injected at first, into each surgical site for hemostasis. The incision at the recipient site started with two vertical elliptical incisions along the cleft margin from the labial side around the labial fistula if present. The labial sulcular incision was completed till two adjacent teeth medial and lateral to the vertical incisions at the area of cleft; then, 2 vertical releasing incisions were performed which extended upwards and backward into the buccal vestibule and finally brought forwards by about 5 mm to give a hockey stick appearance, this allows an easier approximation of the labial flap towards the palatal one without tension after graft placement. After raising full-thickness labial and palatal flaps, exicion of the scar tissue within the bony cleft was achieved. Afterwards, careful dissection of the labial flaps with adequate nasal lining separation and watertight closure of the nasal layer were completed.

For both groups, after gaining access to the anterior iliac crest, a trap door technique was used for cancellous bone chips harvesting. Afterwards, the donor site was closed in layers.

**Fig. 1:**
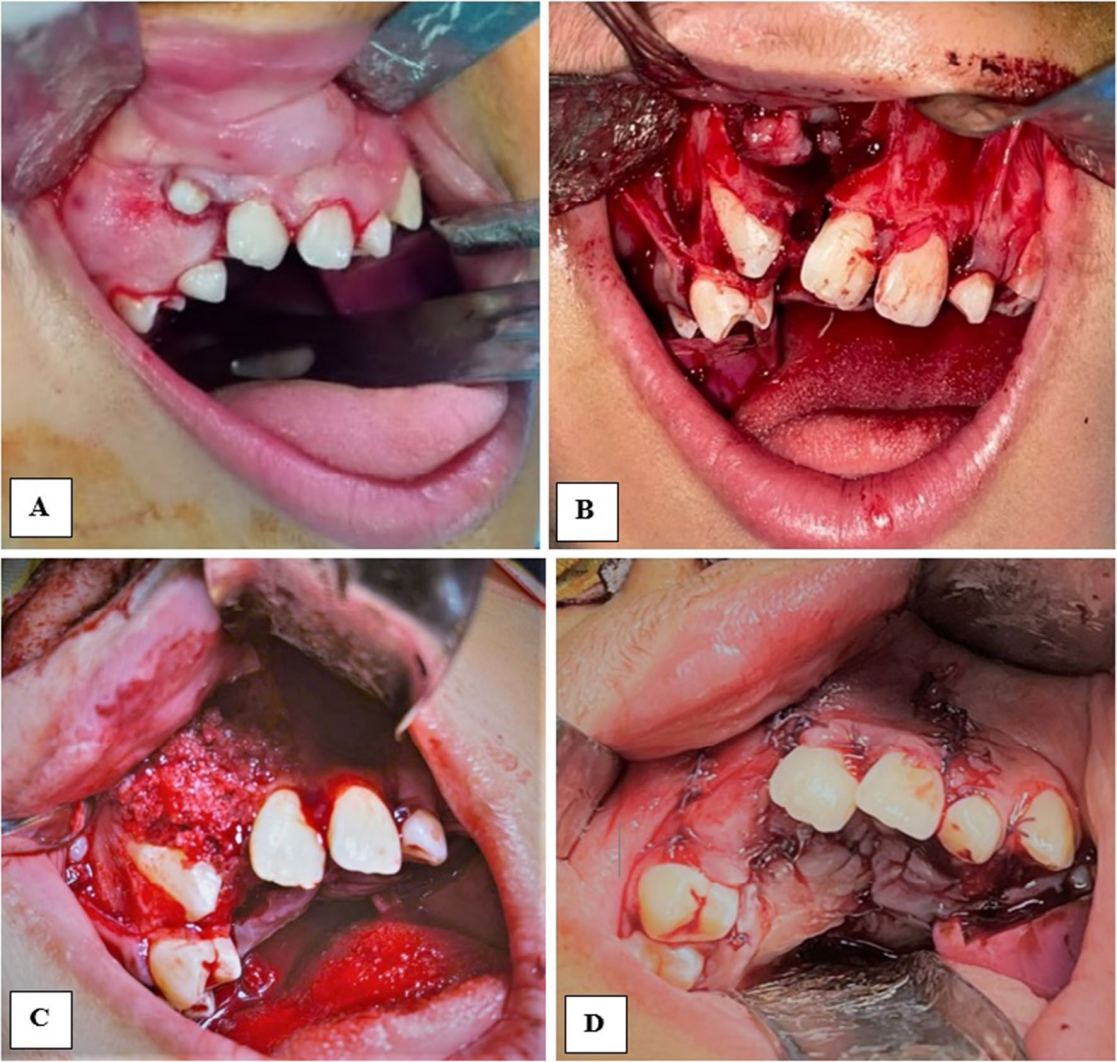
Clinical photographs showing alveolar cleft grafting using conventional bone particles derived from anterior iliac crest (control group)

**Fig. 2:**
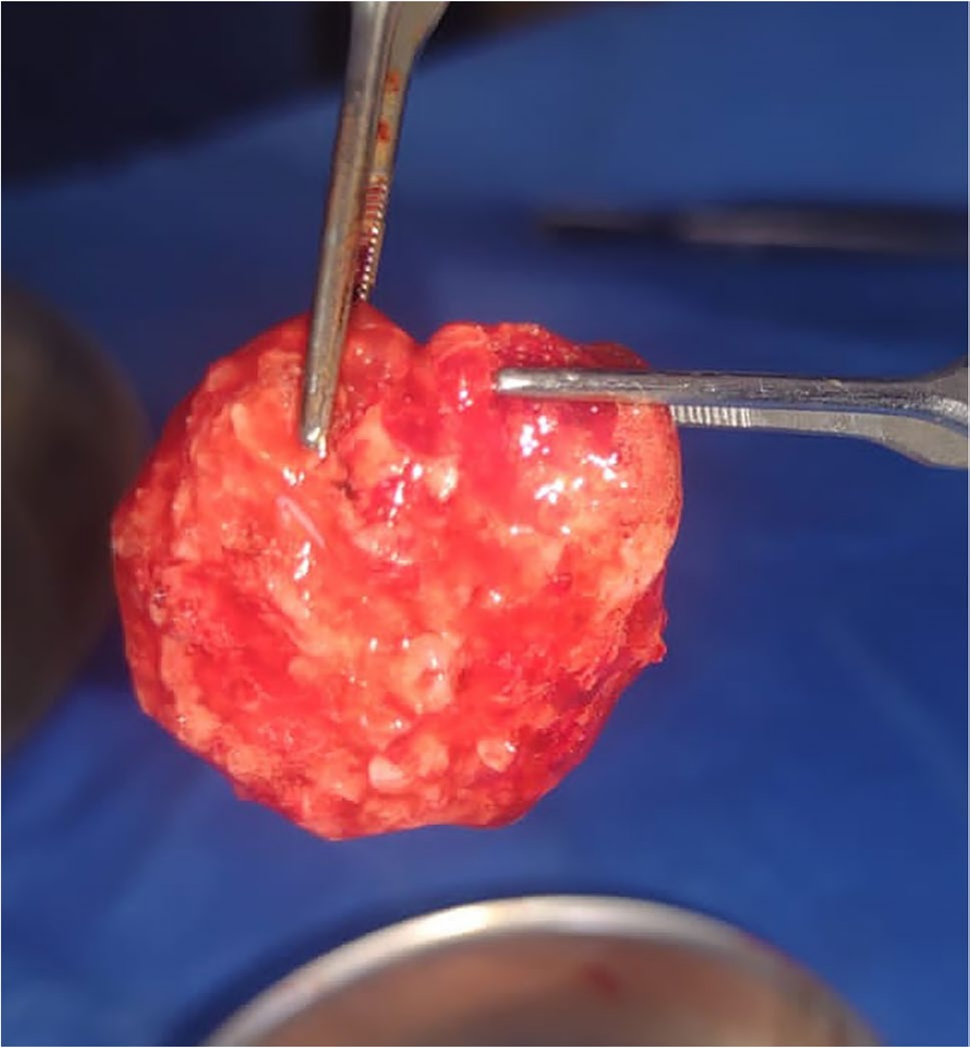
Clinical photographs showing prepared MPM (sticky bone) ready for application within the cleft gap (study group)

**Fig. 3:**
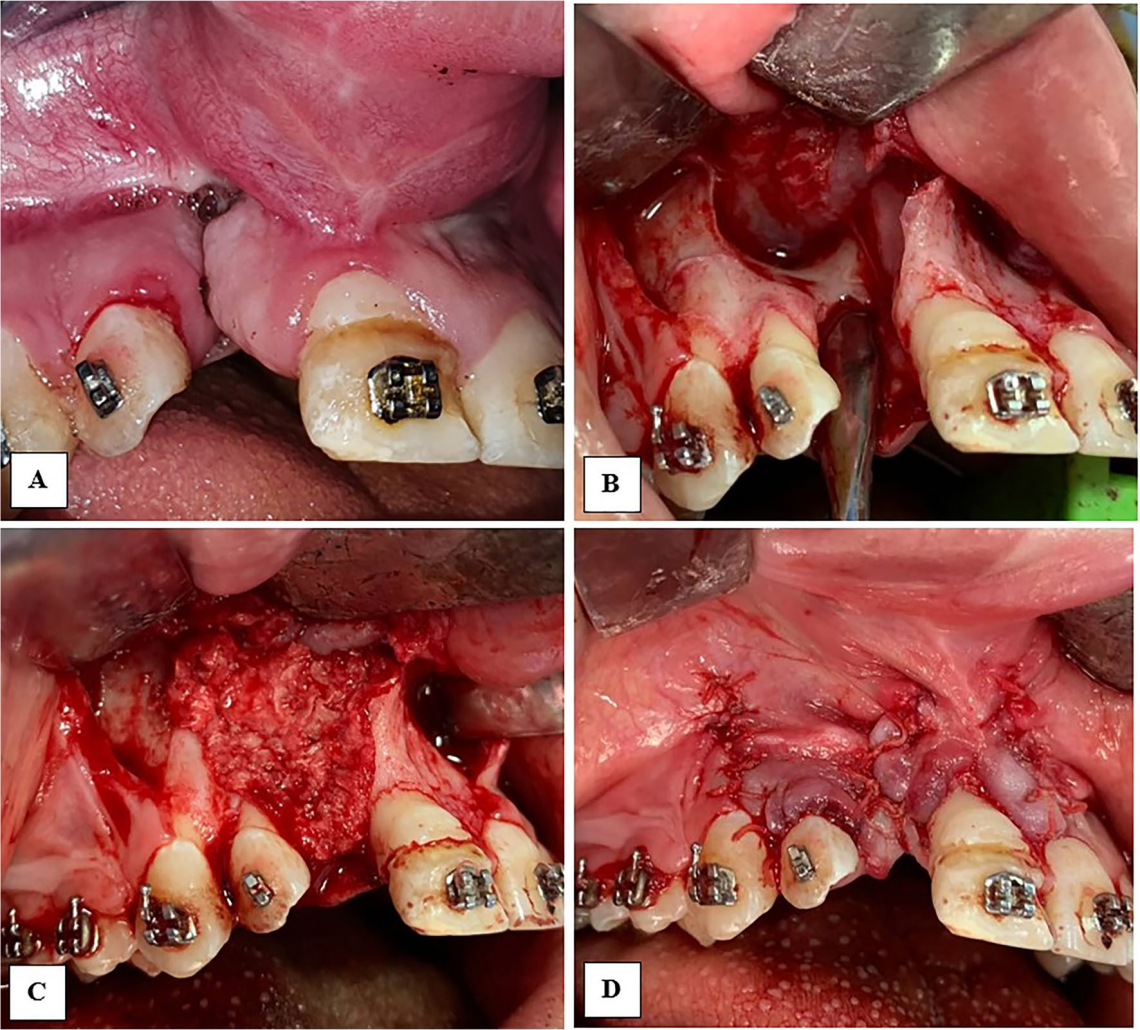
Clinical photographs showing alveolar cleft grafting using MPM (study group)

In the control group, these bone chips were packed directly into the cleft and flap closure followed (Fig. 1). In the study group, blood samples were collected from the patients, placed in 9-mL plastic tubes without anticoagulant (BD Vacutainer) and immediately centrifuged at 2800 rpm for 12 min. The upper yellowish liquid portion of each tube was aspirated using syringes and was combined with the autogenous cancellous bone particles. The mix was stirred immediately using a dentistry probe till fibrin strands began to appear and the mix became like a compact moldable mass (Fig. 2), then this moldable mass was packed and shaped easily within the cleft gap (Fig. 3). For both groups, flap closure and suturing were carried out in a regular fashion using resorbable suture (Coated VICRYL® (polyglactin 4/0) Suture).

### Postoperative follow-up and outcomes

At the donor site, dressing was applied for 48 h while suture removal was done 7 days postoperatively. While at the recipient site, instructions were given to the patient to apply icepacks over the upper lip area for 15 min every hour for 24 h postoperatively and to keep on a soft diet for the first 2 days postoperatively. Moreover, patients were asked to rinse their mouth with warm saline solution starting the second day after surgery for a week Postoperative antibiotic (amoxicillin/clavulanic acid 625 mg^1^ bid for 1 week), non-steroidal anti-inflammatory analgesic (Ibuprofen 200 ml^2^ syrup tid), decongestant nasal drops^3^ tid for 10 days were prescribed. Follow-up was carried out daily during the first week then every month for 6 months to assess the healing of the wounds at the recipient and donor sites and to evaluate whether there is any complication as infection, flap dehiscence, or fistula formation**.**

**Fig. 4:**
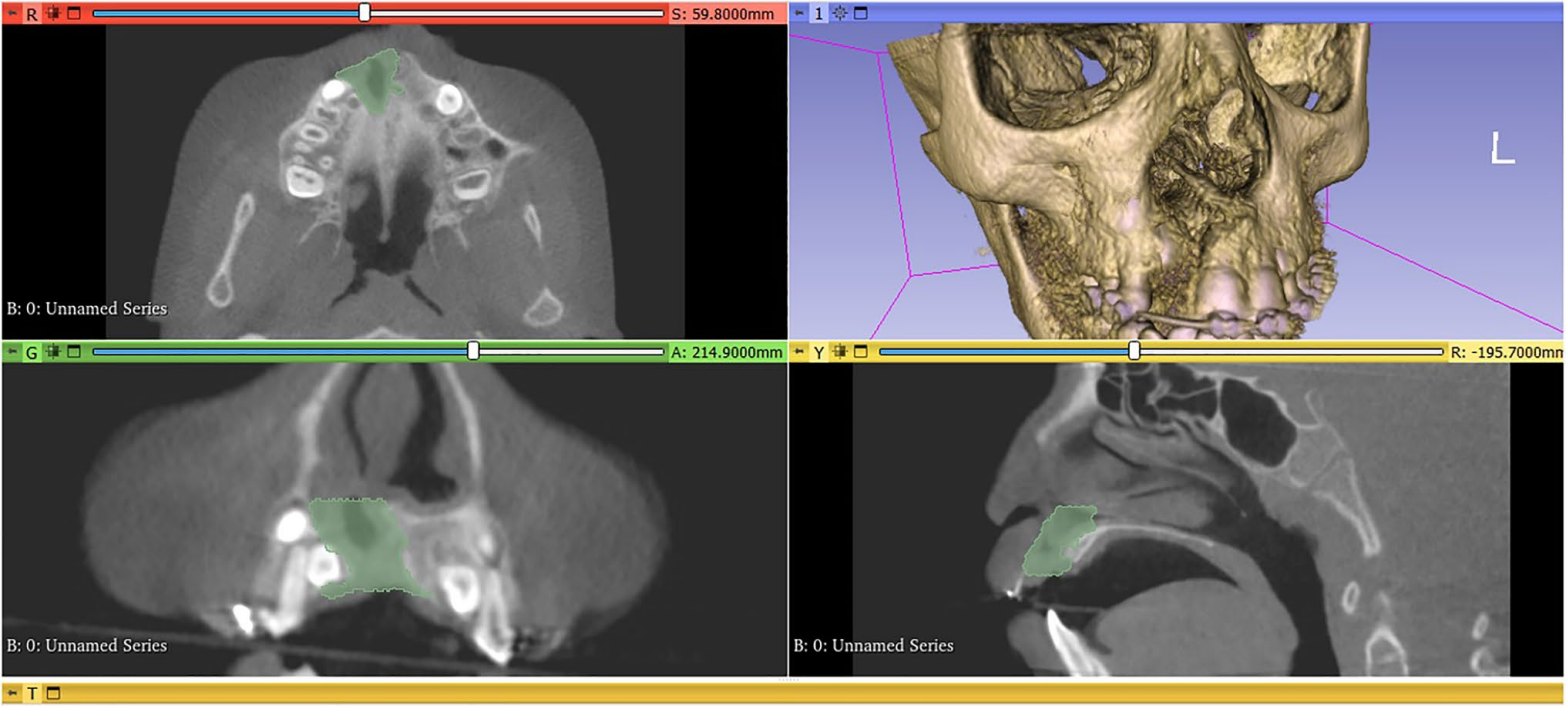
Radiographic photo showing manual segmentation of the cleft area using the (segment statistics) module in 3D Slicer for presurgical cleft volume determination

**Fig. 5:**
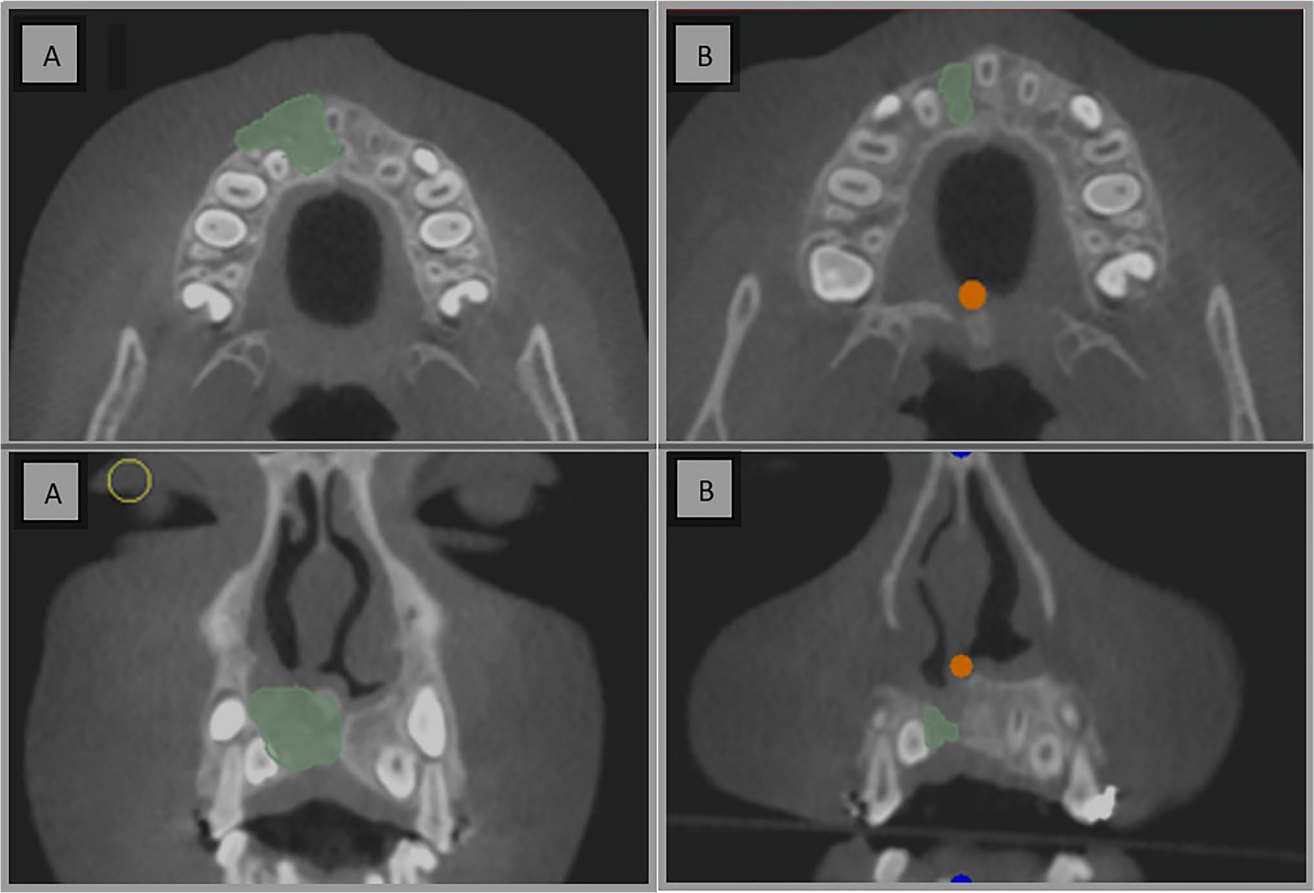
Radiographic photo (axial and coronal views) showing graft volume; **A** Immediately. **B** 6 months postoperatively (control group)

**Fig. 6:**
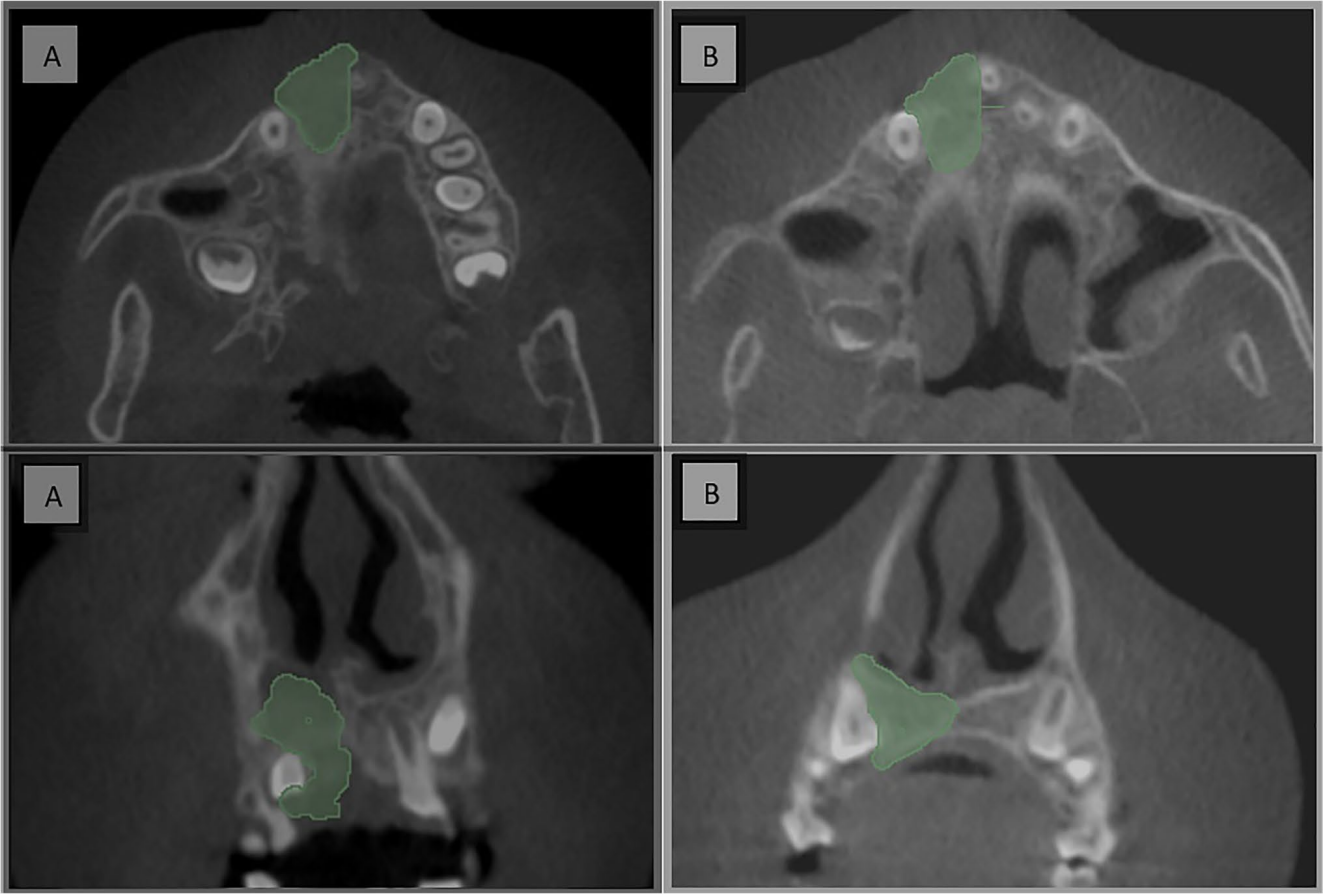
Radiographic photo (axial and coronal view) showing graft volume; **A** Immediately. **B** 6 months postoperatively (study group)

**Fig. 7:**
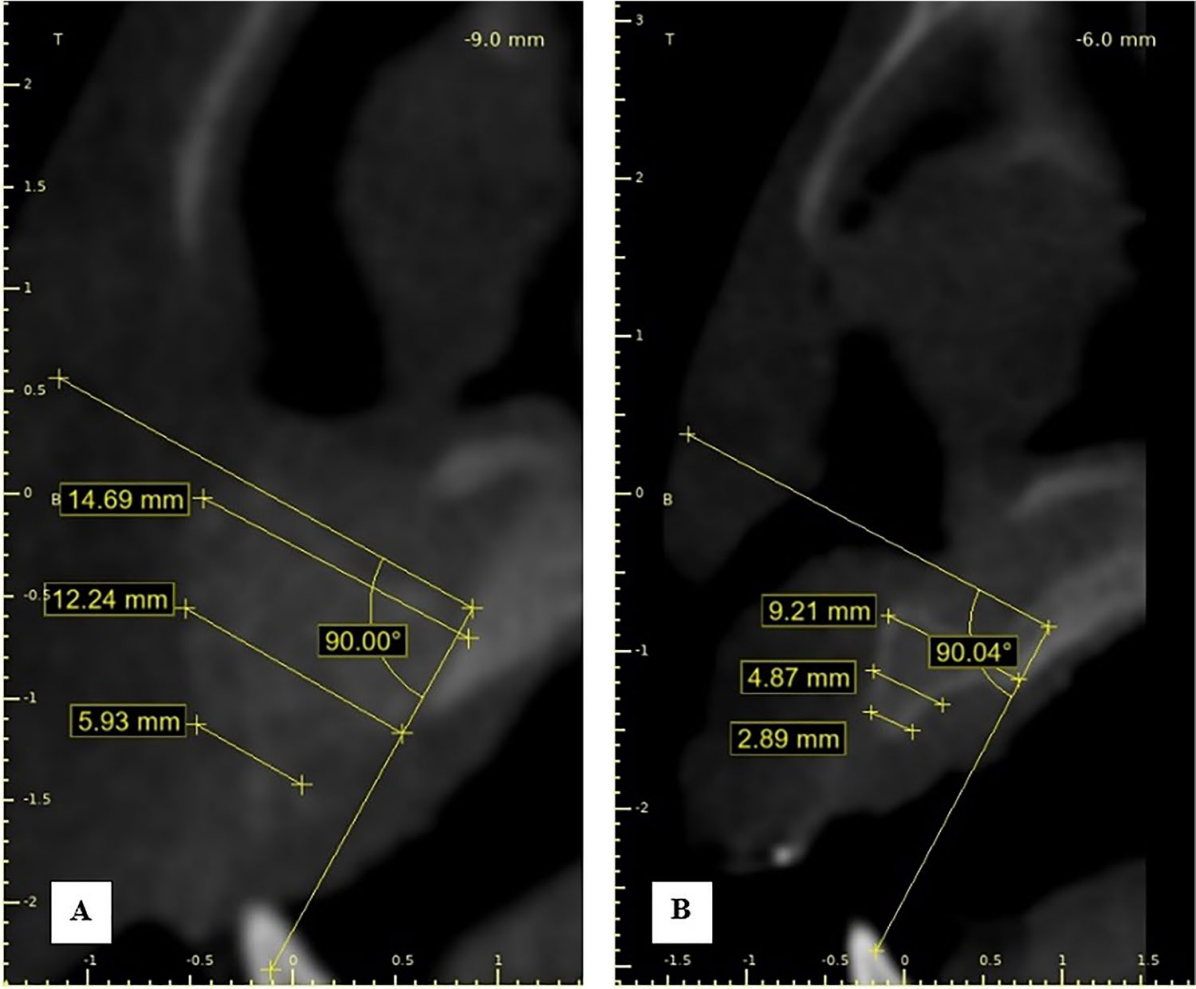
Radiographic photo showing bone width; **A** Immediately. **B** 6 months postoperatively (control group)

**Fig. 8:**
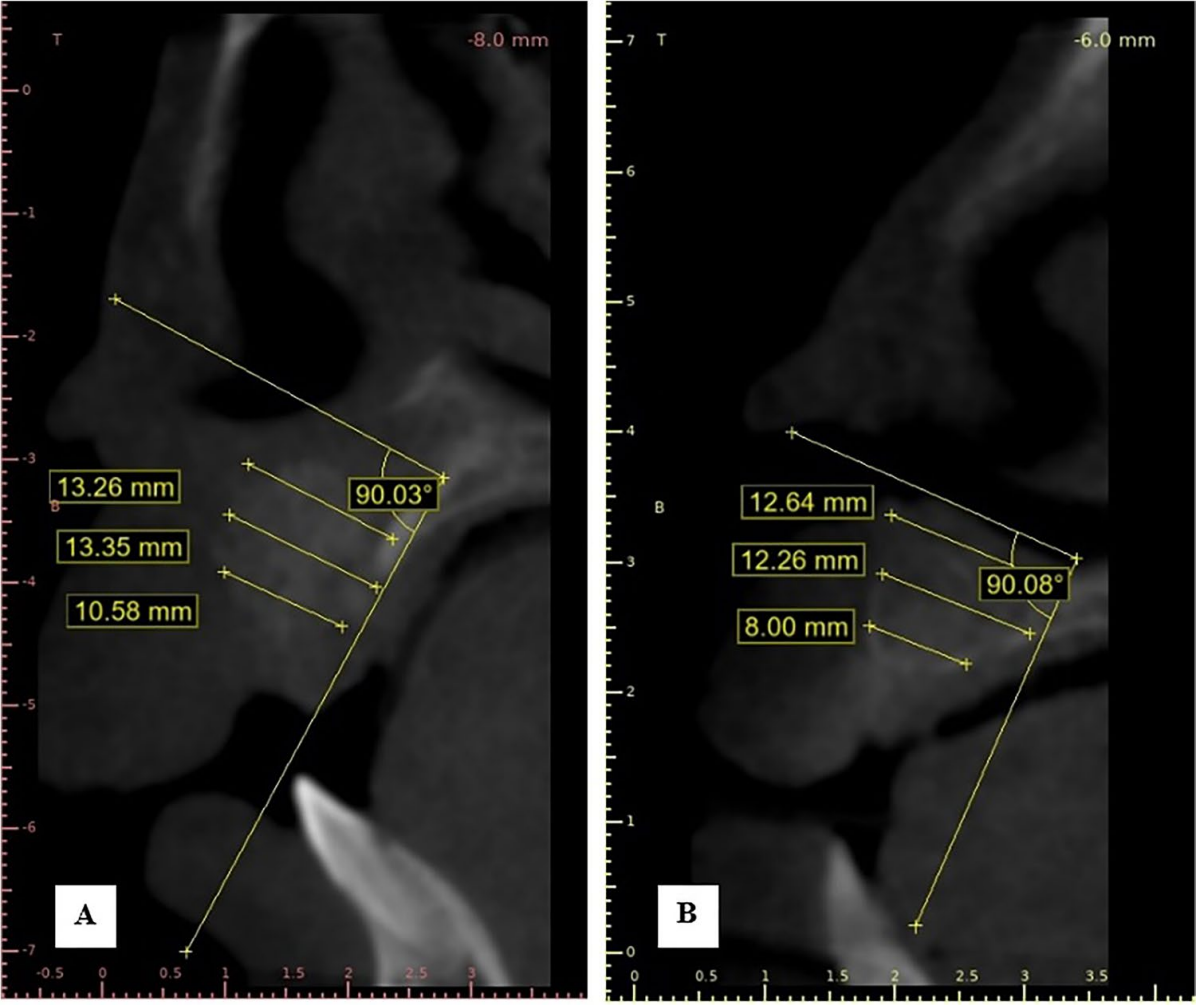
Radiographic photo showing bone width; **A** Immediately. **B** 6 months postoperatively (study group)

**Fig. 9:**
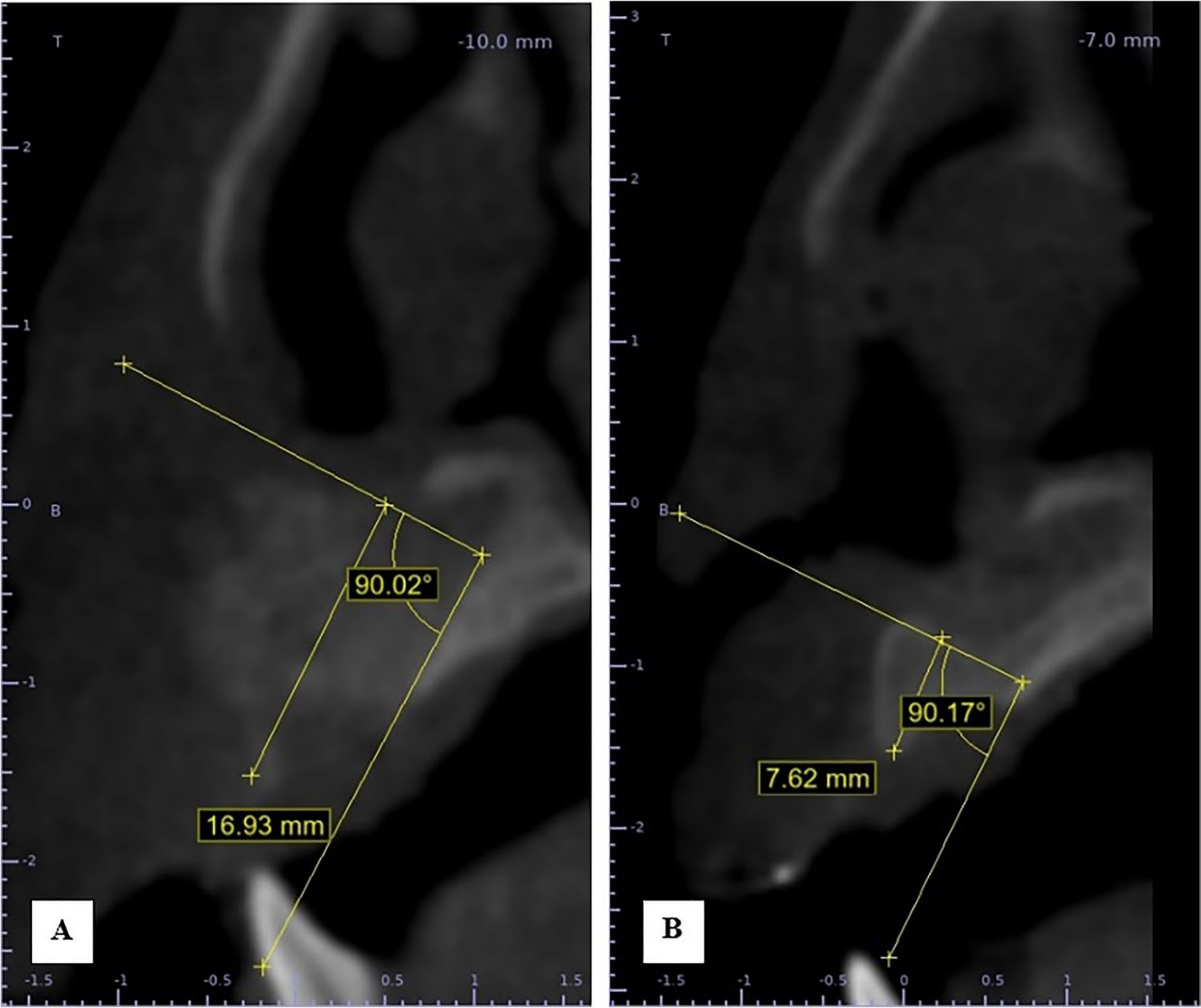
Radiographic photo showing bone height; **A** Immediately. **B** 6 months postoperatively (control group)

**Fig. 10:**
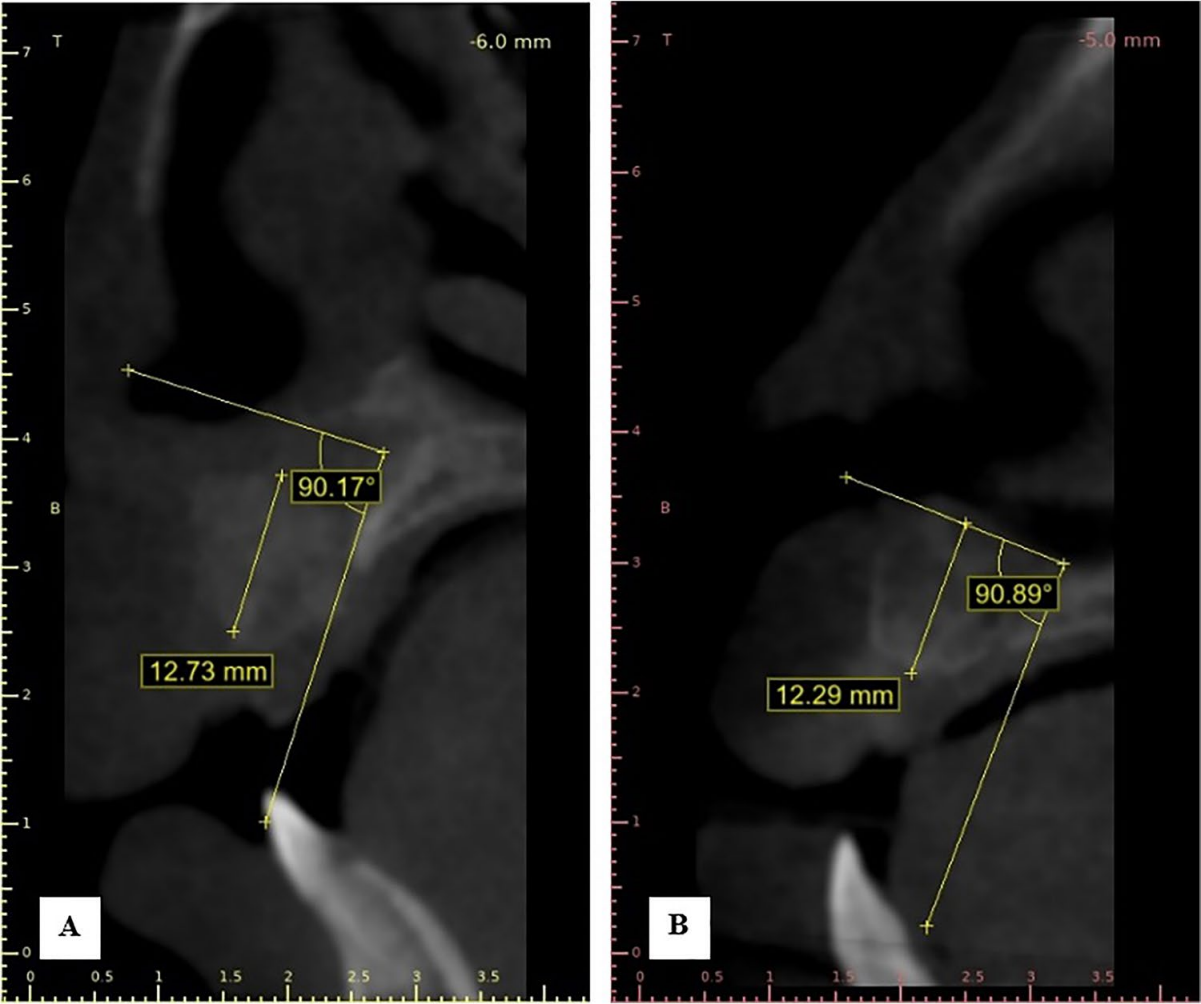
Radiographic photo showing bone height; **A** Immediately. **B** 6 months postoperatively (study group)

Postoperative CBCT scans were taken after one week then after 6 months to assess the graft’s volume, labio-palatal width, and height. Using 3D Slicer (www.slicer.org), volume measurements were carried out using manual segmentation of the cleft area and/or graft by painting slice by slice on each axial image starting from the level of the nasal floor which was determined by the nasal floor of the non-cleft side till the level of crestal bone of the teeth bounding the cleft and finally with the help of the (segment statistics module) of the software, the volume of the segmented area was automatically measured in cm^3^ and mm^3^ [32, 33] (Fig. 4). Immediate and 6 months postoperative graft volume changes were measured and compared (Figs. 5 and 6). Labio-palatal graft width was measured on 3 levels of the cross-sectional cut at the middle of the grafted cleft gap. The three levels on the cross-sectional cut were chosen in relation to the central incisor adjacent to the cleft as follows: level 1 at the CEJ, level 3 at the root apex, and level 2 midway between both. Average of the three measurements was recorded (Figs. 7 and 8). On the same cross-sectional cut, bone graft height was measured through measurement of a line at the center of the grafted area that extended from the nasal floor to the crestal margin of the graft (Figs. 9 and 10). Comparison within the same group between the immediate postoperative and the 6 months postoperative measurements of graft volume, width, and height was done and also between both groups. All measured data were sent for statistical analysis.

### Statistical analysis

Categorical data were presented as frequency and percentage values and were analyzed using Fisher’s exact test. Numerical data were presented as mean and standard deviation (SD) values. Shapiro–Wilk’s test was used to test for normality. Homogeneity of variances was tested using Levene’s test. Data were parametric and showed variance homogeneity so independent and paired *t*-tests were used to analyze inter and intragroup comparisons respectively. The significance level was set at* p* < 0.05 within all tests. Statistical analysis was performed with R statistical analysis software version 4.1.3 for Windows.^4^

## Results

The study was conducted on 10 patients who were equally and randomly allocated to the tested groups. In both groups, there were 3 (60.0%) males and 2 (40.0%) females. Mean age of the cases in the control group was (9.96 ± 2.84) years, while in the study group, it was (10.02 ± 2.03) years (Table 1). Results of intergroup comparisons of demographic data presented in Table 1 showed that there was no significant difference between both groups regarding gender and age (*p* > 0.05).

**Table 1:** Demographic data and volumetric changes of cleft grafting

*Parameter*			*Control*	*MPM*	*Statistic*	*p-value*
*Sex*	***Male***	***n***	3	3	0.00	1
		***%***	60.0%	60.0%		
	***Female***	***n***	2	2		
		***%***	40.0%	40.0%		
*Age (years)*	***Mean* ± *SD***		9.96 ± 2.84	10.02 ± 2.03	0.04	0.970
Pre-surgical cleft volume	**Volume (cm**^**3**^**) (Mean ± SD)**		2.08 ± 0.65	1.88 ± 0.65	0.50 (t-value)	0.631
Post-surgical percentage of graft volume reduction (immediate versus 6 months)	**Volume change** **(%) (Mean ± SD)**		48.91 ± 4.73	19.64 ± 2.93	11.84 (t-value)	< 0.001*

^*^significant (*p* < 0.05)

The mean cleft volume of the cases in the control group was 2.08 ± 0.65 cm^3^ while in the study group was 1.88 ± 0.65 cm^3^ (Table 1). Results of intergroup comparison of cleft volume presented in Table 1 showed that there was no significant difference between cleft volumes measured in both groups before surgery (*p* = 0.631) and the cleft was extended labio-palatally in all patients of both groups, while the percentage of post-surgical graft volume reduction measured in the control group immediately versus 6 months postoperatively was (48.91 ± 4.73%) which is statistically significantly higher than that in MPM group which was (19.64 ± 2.93%) (*p* < 0.001) (Table 1).

Results of inter and intragroup comparisons of bone width presented in Table 2 showed that immediately post-operatively, there was no significant difference between both groups (*p* = 0.467). While after 6 months, MPM group had significantly higher bone width than the control group (*p* = 0.034). For the control group, there was a significant decrease of bone width after 6 months (*p* = 0.013), while for MPM group, there was no significant difference between bone width values measured immediately postoperatively and after 6 months (*p* = 0.751). The control group had significantly higher width decrease after 6 months in comparison to MPM group (*p* < 0.05).

**Table 2:** Inter and intragroup comparisons of bone width

*Interval*	*Bone width (mean* ± *SD)*		*t-value*	*p-value*
	*Control*	*Study*		
*Immediately post-operative (mm)*	12.75 ± 2.50	11.77 ± 1.40	0.76	0.467
*After 6 months (mm)*	7.94 ± 1.24	11.49 ± 2.84	2.55	0.034*
*t-value*	4.28	0.34		
*p-value*	0.013*	0.751		
*Difference (mm)*	4.80 ± 2.51	1.06 ± 0.15	3.33	0.011*
*Percentage change (%)*	36.11 ± 14.59	8.47 ± 3.54	4.12	0.003*

^*^significant (*p* < 0.05)

Results of inter and intragroup comparisons of bone height presented in Table 3 showed that immediately postoperatively, there was no significant difference between both groups (*p* = 0.324). While after 6 months, MPM group had significantly higher bone height than the control group (*p* = 0.030). For both groups, there was a significant decrease of bone height after 6 months (*p* < 0.05). The control group had significantly more height reduction after 6 months in comparison to MPM group (*p* < 0.05).

**Table 3:** Inter and intragroup comparisons of bone height

*Interval*	*Bone height (mean* ± *SD)*		*t-value*	*p-value*
	*Control*	*Study*		
*Immediately post-operative (mm)*	12.24 ± 1.42	11.39 ± 1.15	1.05	0.324
*After 6 months (mm)*	8.53 ± 0.37	10.27 ± 1.44	2.62	0.030*
*t-value*	6.18	3.52		
*p-value*	0.003*	0.024*		
*Difference (mm)*	3.71 ± 1.34	1.11 ± 0.71	3.83	0.005*
*Percentage change (%)*	29.64 ± 8.06	9.91 ± 6.12	4.36	0.002*

^*^significant (*p* < 0.05)

## Discussion

Secondary alveolar cleft grafting aims to create a bone bridge connecting the two maxillary bony segments and hence stabilizing the maxillary arch, closure of oronasal fistula and to create a sound bone foundation for the future eruption of the cleft teeth and/or for implant replacement of missing cleft teeth.

Autogenous cancellous bone graft harvested from the anterior iliac crest is considered the “gold standard” for alveolar cleft reconstruction as it is easy to access, offers large quantities of autogenous bone which contain large amount of osteogenic cells that promote osteogenesis, and it has osteo-inductive capability as it contains viable cells and growth factors that ensure total consolidation and incorporation with the surrounding maxillary bone, but the lack of positional stability and the subsequent high resorption rate of the iliac spongiosa decrease its bone grafting quality and might result in the need for another grafting maneuver [6, 7, 28].

To improve the bone grafting outcome, a number of qualities need to be enhanced, like space maintenance, scaffolding, and the stability of the graft. Mineralized plasmatic matrix (MPM) preparation shows the simplicity of the PRF protocol while yielding a liquid platelet/fibrin concentrate that can bind to bone, and it is the only natural and autogenous product that offers stability to the bone chips and this offers the mouldability and positional stability through chemical stabilization of the bone graft and preservation of its shape with subsequent “in situ” immobilization.

The MPM is prepared by centrifugation of collected autogenous blood samples using plain tubes without neither anticoagulant or clot activator. At the end of the centrifugation, the superior liquid part of the tube contains fibrinogen, platelets, and monocytes. This liquid will be collected and added to the autogenous bone graft chips, once this plasma is in contact with the bone graft, the activation will start and the fibrinogen will transform into the fibrin network.

This graft manipulation is very important because it induces physical changes to the harvested bone particles to become homogenous, easy-to-handle, and of moldable consistency in addition to bio-chemical changes as it enriches the graft with the fibrin that acts as fibrin glue and by that, the whole mass of the MPM graft becomes moldable and stable in place. In addition to that, the MPM contains cells such as platelets which add growth factors and cytokines in addition to monocytes that play an important role in the regulation of the natural bone morphogenic protein (BMP). Thus MPM, based on its structure, should be considered whenever a bone grafting procedure is attempted [30, 31, 34].

Similar to the results of the current study, Marukawa et al. (2011) found that the use of PRP yielded better results for decreasing the resorption of the height and width of the bone graft as compared to autogenous cancellous bone alone [7]. Also, Nadon et al. (2015) had a complete cleft closure using MPM in 10 of 10 patients (100%), with all patients having stable bone bridges in the cleft area and stable teeth adjacent to the clefts in their study [30].

Based on the literature, authors believed that the MPM offered stability for the graft as MPM contains platelets and fibrin concentrate in a liquid state; these materials can harden and bound to bone particles giving a moldable bone mass that was easy to shape which is responsible for graft height and width stability over time, So the 6-month outcomes of MPM were excellent regarding bone graft stability that was reflected positively in term of maintained graft bone width and height. On the other hand, the control group bone particulates stability is highly affected by the chewing forces, food impinging over the overlying mucosa and the movement of the constricted and scarred lip in some patients which results in reduced graft height and width after a period of time as it lakes the main cause for graft success which is graft stability. Also, the percentage of bone volume changes showed that graft instability in the control group led to reduction of 48.9% of graft volume, while in the MPM group, the reduction of graft volume was limited to 19.6% owing to the graft stability that support the actual mechanism of the MPM grafting technique also the enriched growth factors that limit the graft resorption and enhanced its consolidation reflected positively in term of maintained graft volume width and height [30].

The most important advantage of the MPM — against the gold standard procedure — is the incorporation of bone graft particles inside the fibrin network, which grants the MPM positional stability by stabilizing the bone particles, preserving their shape and volume with subsequent “in situ” immobilization of the components of grafting materials [30].

## Conclusion

Alveolar cleft grafting using MPM (study group) showed greater stability in terms of graft volume, labio-palatal bone width, and bone height as compared to the gold standard particulate cancellous bone derived from anterior iliac crest alone (control group).

## Data Availability

All data are available whenever requested.

## Footnotes

1 Augmentin, GlaxoSmithKlein, Egypt.

2 Brufen,Abott laboratories, Egypt.

3 Afrin, Shering Plough, Egypt.

4 R Core Team (2022). R: A language and environment for statistical computing. R Foundation for Statistical Computing, Vienna, Austria. URL https://www.R-project.org/.
